# Persistent Mitochondrial Hyperfusion Promotes G2/M Accumulation and Caspase-Dependent Cell Death

**DOI:** 10.1371/journal.pone.0091911

**Published:** 2014-03-14

**Authors:** Laura M. Westrate, Aaron D. Sayfie, Danielle M. Burgenske, Jeffrey P. MacKeigan

**Affiliations:** 1 Laboratory of Systems Biology, Van Andel Research Institute, Grand Rapids, Michigan, United States of America; 2 Van Andel Institute Graduate School, Grand Rapids, Michigan, United States of America; Roswell Park Cancer Institute, United States of America

## Abstract

Cancer cells have several hallmarks that define their neoplastic behavior. One is their unabated replicative potential that allows cells to continually proliferate, and thereby contribute to increasing tumor burden. The progression of a cell through the cell cycle is regulated by a series of checkpoints that ensures successful transmission of genetic information, as well as various cellular components, including organelles and protein complexes to the two resulting daughter cells. The mitochondrial reticulum undergoes coordinated changes in shape to correspond with specific stages of the cell cycle, the most dramatic being complete mitochondrial fragmentation prior to cytokinesis. To determine whether mitochondrial fission is a required step to ensure proper mitochondrial segregation into two daughter cells, we investigated the importance of mitochondrial dynamics to cell cycle progression. We found that mitochondrial hyperfusion promotes a defect in cell cycle progression characterized by an inability for cells to exit G2/M. Additionally, extended periods of persistent mitochondrial fusion led to robust caspase-dependent cell death. The cell death signals were coordinated through activation and cleavage of caspase-8, promoting a potent death response. These results demonstrate the importance of mitochondrial dynamics in cell cycle progression, and that inhibiting mitochondrial fission regulators may provide a therapeutic strategy to target the replicative potential of cancer cells.

## Introduction

Mitochondria play critical roles in meeting the bioenergetics needs of the cell, which include the generation of cellular ATP through oxidative phosphorylation [1]. Maintaining mitochondrial function is therefore a priority for cells. The evolutionarily conserved process of mitochondrial fission and fusion has proven to be an important mechanism by which mitochondria maintain function and respond to changing cellular demands. Many tumors, however, have a glycolytic metabolic profile that is no longer dependent on the mitochondria as the source for their metabolic and energetic needs [2], [3]. Despite this, mitochondria in cancer cells are highly active and dynamic, suggesting an important role for mitochondrial fission and fusion in cancer biology. Mitochondrial fission and fusion is controlled by a series of well conserved GTPases from the dynamin family [1]. Mitochondrial fusion of the outer mitochondrial membrane (OMM) is initiated through interactions between two transmembrane GTPases, mitofusin-1 (Mfn1) and mitofusin-2 (Mfn2), while fusion of the inner mitochondrial membrane (IMM) is regulated by a third GTPase, optic atrophy 1 (OPA1) [4], [5], [6], [7]. A fourth GTPase, dynamin related protein 1 (Drp1) regulates mitochondrial fission and is recruited from the cytosol to the mitochondrial by a series of OMM proteins (mitochondrial fission factor, Mff; fission 1, Fis1; mitochondrial elongation factor 49, MiD49; mitochondrial elongation factor, MiD51; or endophilin B1) [8], [9]. Influenced by their surrounding cellular environment, mitochondrial morphology is not only important for maintaining mitochondrial function, but has recently been marked as an important cellular feature for the completion of biological processes, including cellular proliferation and apoptosis [10], [11], [12].

Recently, mitochondria have been shown to undergo dramatic remodeling prior to cell division [11]. Mitotic cell division of eukaryotic cells can be divided into four major stages including a growth stage (G1), a DNA replication stage (S), a secondary growth stage (G2), and cell division (M) [13]. Quantitative assessment of mitochondrial morphology throughout the various stages of the cell cycle reveals that mitochondria fuse to form a large, hyperfused network at the G1-S transition before undergoing coordinated fragmentation in G2/M [11]. While in its hyperfused state, the mitochondrial network is electrically continuous, resulting in greater ATP output which may be required to promote transition of cells through S [11]. Additionally, mitochondrial hyperfusion can result in a buildup of cyclin E, which at the G1-S transition, is responsible for the initiation of DNA replication and further commitment of the cell to undergo mitosis [11]. Loss of Drp1, the GTPase involved in regulating mitochondrial fission, resulted in G2/M accumulation [12]. This result suggests that mitochondrial fission is necessary for continued progression through the cell cycle following entrance of the cell into S phase [12].

Given the observation that mitochondria fragment prior to cell division, we predicted that the shape of the mitochondria plays an important role in the ability for cells to progress through the cell cycle. Here, we investigate the role of mitochondrial fission machinery in cell cycle progression. We found that when mitochondria are maintained in a state of fusion, cell cycle progression is significantly delayed and cells accumulate in G2/M [12]. This cell cycle defect is recapitulated upon knockdown of key mitochondrial fission regulators, Drp1 or Mff, supporting the finding that mitochondrial fission is a requisite step for cell division. This suggests that mitochondrial fission may be an important mechanism to ensure proper segregation of mitochondria into the two daughter cells. Surprisingly, loss of either Drp1 or Mff results not only in a G2/M cell cycle defect, but also in potent induction of caspase 8 dependent cell death. Taken together, these results demonstrate the important role mitochondrial fission and fusion play in cell cycle progression and cell survival. Given the unabated replicative potential of cancer cells, therapeutic strategies targeting mitochondrial dynamics may provide a novel means to target cancer’s neoplastic behavior.

## Materials and Methods

### Cell Culture and RNAi Transfection

U2OS cells (ATCC) were cultured in normal cell maintenance media containing McCoy’s 5A (Life Technologies) supplemented with 10% fetal bovine serum (CellGro). For RNAi transfection experiments, U2OS cells were seeded in 6-well plates at a density of 7.5×10^4^ cells per well for 24 hours before being transfected with either control (non-targeting) siRNAs or siRNAs targeted against mitochondrial fission regulators, Drp1 and Mff: (Qiagen Drp1 #1 S104320092, Drp1 #2 SI04202464, Mff #1 SI04320386, Mff #2 SI04374174) at a final concentration of 50 nM using 3 μl Oligofectamine (Life Technologies) in 0.2 ml Opti-MEM (Life Technologies) and 0.8 ml normal cell maintenance media. Transfection wells were supplemented with an additional 1 ml of normal cell maintenance media 4 hours following the addition of the transfection reagent. Drp1 siRNA SI04320092 and Mff siRNA SI04320386 resulted in the highest level of mitochondrial fusion and were subsequently used to assess the cellular consequences of mitochondrial fusion for both cell cycle and cell death experiments. Knockdown was measured by qRT-PCR using specific primers against Drp1 and Mff and the endogenous hypoxanthine phosphoribosyltransferase 1 (HPRT) control. Relative copy number was determined using the delta-delta Ct method for control, Drp1, and Mff siRNA samples [14].

### Cell Cycle Analysis

U2OS cells were seeded in 6-well plates at a density of 7.5×10^4^ cells per well and transfected for 48 hours with siRNAs directed against Drp1 and Mff, as described above. Adherent cells were collected and resuspended in ice-cold 70% ethanol. Samples were permeabilized at −20°C overnight. Propidium iodide (PI; Sigma) was prepared as a 1 mg/ml stock solution in H_2_O. RNase A was prepared as a 10 mg/ml stock solution in 10 mM sodium acetate, pH 5.0−5.2. DNase was inactivated by heating the RNase A solution to 100°C for 15 minutes before adjusting to 100 mM Tris, pH 7.4 by adding 0.1 volume of 1 M Tris-HCl. Samples were washed 1x with PBS to remove residual ethanol and resuspended in a PI working stock (20 μg/ml PI, 2 mg DNase free RNase A added to PBS) for 10 minutes before being analyzed for DNA content on a FACSCaliber flow cytometer (BD Biosciences) using the FL2 channel to determine PI fluorescence. At least 10,000 events were captured for each sample. Cell Cycle profiles were analyzed using Modfit LT following the manufacturer’s protocol to determine the percentage of cells in G1, S and G2/M, error was determined by calculating the standard deviation of replicate experiments.

### Cell Synchronization

U2OS cells were synchronized at late G1, early S by a double thymidine block. U2OS cells were seeded in 6-well plates and transfected as described above. The first thymidine block was started 8 hours following siRNA transfection by the addition of thymidine (Sigma) to wells at a final concentration of 2 mM for 16 hours. Cells were then washed 1x with PBS and cultured in normal cell maintenance media for 8 hours followed by a second 16 hour thymidine (2 mM) block. Cells were released from the double thymidine block into normal cell maintenance media and collected every 4 hours following release. Cells were put into their first thymidine block exactly 8 hours following siRNA transfection to ensure that the 0 hour release collection was timed at 48 hours post knockdown. Samples were fixed in ice-cold 70% ethanol and permeabilized at −20°C overnight. Cell cycle analysis was performed following PI incorporation as described above. Cell Cycle profiles were analyzed using Modfit software to determine percentage of cells in G1, S and G2/M. Error was determined by calculating the standard deviation of the replicate experiments.

### Immunofluorescence

A monoclonal population of U2OS cells expressing mito_EYFP (U2OS_mitoEYFP) was generated following selection with 500 μg/ml G418. U2OS_mitoEYFP cells were seeded onto 1.5 coverglass, 35 mm glass bottom culture dishes (MatTek) at a density of 7.5×10^4^ cells per well 72 hours prior to imaging. Mitochondrial morphology was altered through knockdown of mitochondrial fission regulators Drp1 and Mff. All images were taken in an environmental chamber, 48 hours after knockdown on a Nikon Ti Eclipse fluorescent microscope which maintained a humid environment of 37°C and 5% CO_2_. Imaging of U2OS_mitoEYFP was performed using a 60x oil immersion objective (NA 1.4) in the FITC channel (25% lamp power; ND4 and ND8 neutral density filters; 100 ms exposure).

For extended live cell imaging movies, U2OS_mitoEYFP were seeded in 35 mm, #1.5 glass bottom, 4 chambered dishes (MatTek) and were transfected with siRNA targeting Drp1 and Mff for 48 hours before the start of the time-lapse movie. Cells were imaged in the FITC channel using a 40x dry objective. Fields of view were chosen, and NIS Elements software (Nikon) was set to image each position every 10 minutes for 18 hours using the Perfect Focus System (PFS) to maintain the original focal plane. Individual cells were manually tracked throughout the entire time series to determine whether cells underwent mitotic cell division, cell death, or neither. If cells moved out of the field of view at any point during the time series, they were no longer considered part of the data set. Cell death was identified as cells undergoing classical morphological characteristics of apoptosis, such as plasma membrane blebbing and nuclear condensation.

To obtain 3D-reconstruction of dividing cells, live cell imaging was performed on a Nikon A1Rsi Confocal System. U2OS_mitoEYFP cells were seeded in 1.5 coverglass, 35 mm glass bottom culture dishes as described above in 2 ml normal cell maintenance media for 48 hours prior to imaging. Cells were then placed on the stage of the Nikon A1 microscope within a Okolab stage-top incubation system that maintained a humid environment of 37°C and 5% CO_2_. Cells were imaged following simulation with the 488 nm diode laser using a 60x oil (NA 1.4) objective. A z-stack of 6 μm in width (made up of 1 μm stacks) was set up to capture the entire mitochondrial reticulum through cytokinesis. Fields of view were chosen and images were taken every 10 minutes for 11 hours. The focal plane was maintained throughout the length of the movie using PFS. NIS-Elements software was used to perform 3D reconstructions and visualize the mitochondrial reticulum throughout cellular division.

### Cell Death and Apoptosis Assays

Activation of apoptosis through the detection of cleaved caspases was performed on the FACSCaliber flow cytometer according to the protocol previously described [15]. Antibodies towards cleaved caspase-3 (Cell Signaling Technologies) and cleaved caspase-8 (Cell Signaling Technologies) were used to detect apoptotic induction. Necrostatin (Enzo Life Sciences) and zVAD (Millipore) were used at final concentrations of 10 μM and 20 μM, respectively, in cell culture medium prior to PI exclusion assays. Treatment of necrostatin or zVAD was initiated 24 hours after siRNA transfection, and continued every 24 hours until the endpoint of the assay. PI exclusion assays were performed by collecting cells, washing in PBS, and incubating in samples in 2 μg/ml PI for 5 minutes before analyzing for PI positivity as a marker for cell death. At least 10,000 events were captured per experimental condition. Data were analyzed using CellQuest software (BD Biosciences) and error bars plotted represent standard deviation of replicate conditions.

## Results

### Mitochondria undergo architectural changes during progression through the cell cycle

**Figure 1:**
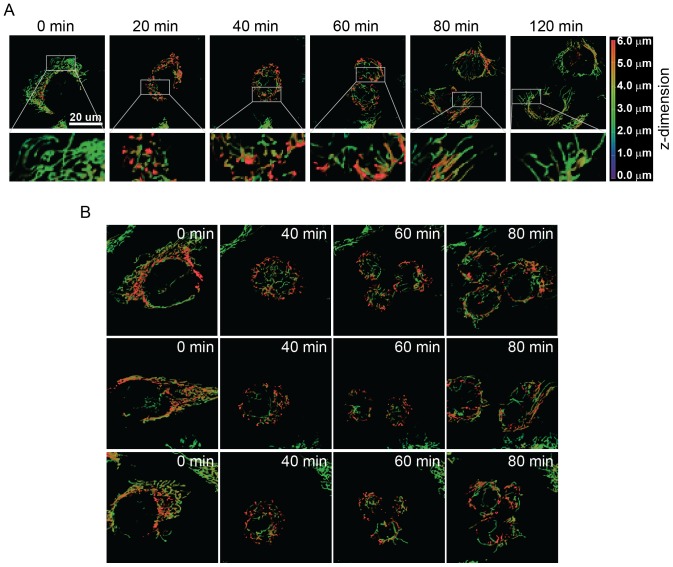
Mitochondrial Fragmentation Prior to Cytokinesis. (A) Mitochondria were tracked through mitotic division events in U2OS_mitoEYFP cells by confocal microscopy. An example cell is shown representing a 3D reconstruction of a 6 μm z-stack (1 μm thick slices) that has been re-colored according to the depth (see color coded legend) of the fluorescent signal within the z-stack. Insets are 3.5x magnifications of boxed regions. (B) Example cells are shown which demonstrate similar morphological alterations in mitochondrial reticulum throughout multiple mitotic cell divisions.

Mitochondria are in constant flux, the reticulum transitioning from states of heavily fused networks to individual punctate fragments spread throughout the cytosol. Alterations to mitochondrial shape are coordinated through two opposing processes, mitochondrial fission and fusion, often in response to cellular demands [1]. Given that mitochondrial morphology is altered in preparation for mitotic cell division [11], we proposed that mitochondrial fission and fusion are important regulatory processes in determining the success of a cell division event by ensuring segregation of the mitochondrial reticulum to the resulting daughter cells. We monitored mitochondrial morphology throughout mitotic cell division using a monoclonal osteosarcoma cell line expressing a mitochondrial-targeted enhanced yellow fluorescent protein (U2OS_mitoEYFP). The EYFP construct is fused to subunit VIII of cytochrome c oxidase (complex IV) and provides a specific means to monitor changes in mitochondrial morphology in real time [16]. Using live cell imaging, we tracked cells progressing through the cell cycle and observed dramatic remodeling of the mitochondrial reticulum during cell division (Figure 1A). Because the mitochondrial reticulum of a dividing cell often moves in and out of the focal plane as the cell begins to enter mitosis [17], [18], we acquired stacks of mitochondrial images (6 μm total depth) along the z-axis of cells. These stacks were then volumetrically rendered following 3D image reconstruction to allow us to track morphological changes of the entire reticulum. U2OS_mitoEYFP cells were tracked for 11 hours with images taken every 10 minutes. We found that prior to cytokinesis, or about 40 minutes before the formation of the resulting daughter cells, mitochondria condense and undergo widespread fragmentation (Figure 1A). This form of mitochondrial remodeling was consistent in all mitotic cells that we tracked (15 cellular division events from over 100 cells), suggesting that the alterations in mitochondrial morphology, specifically through mitochondrial fission, is an important step for preparation of the mitochondrial reticulum prior to cellular division (Figure 1B).

### Persistent mitochondrial hyperfusion results in delayed cell cycle progression

**Figure 2:**
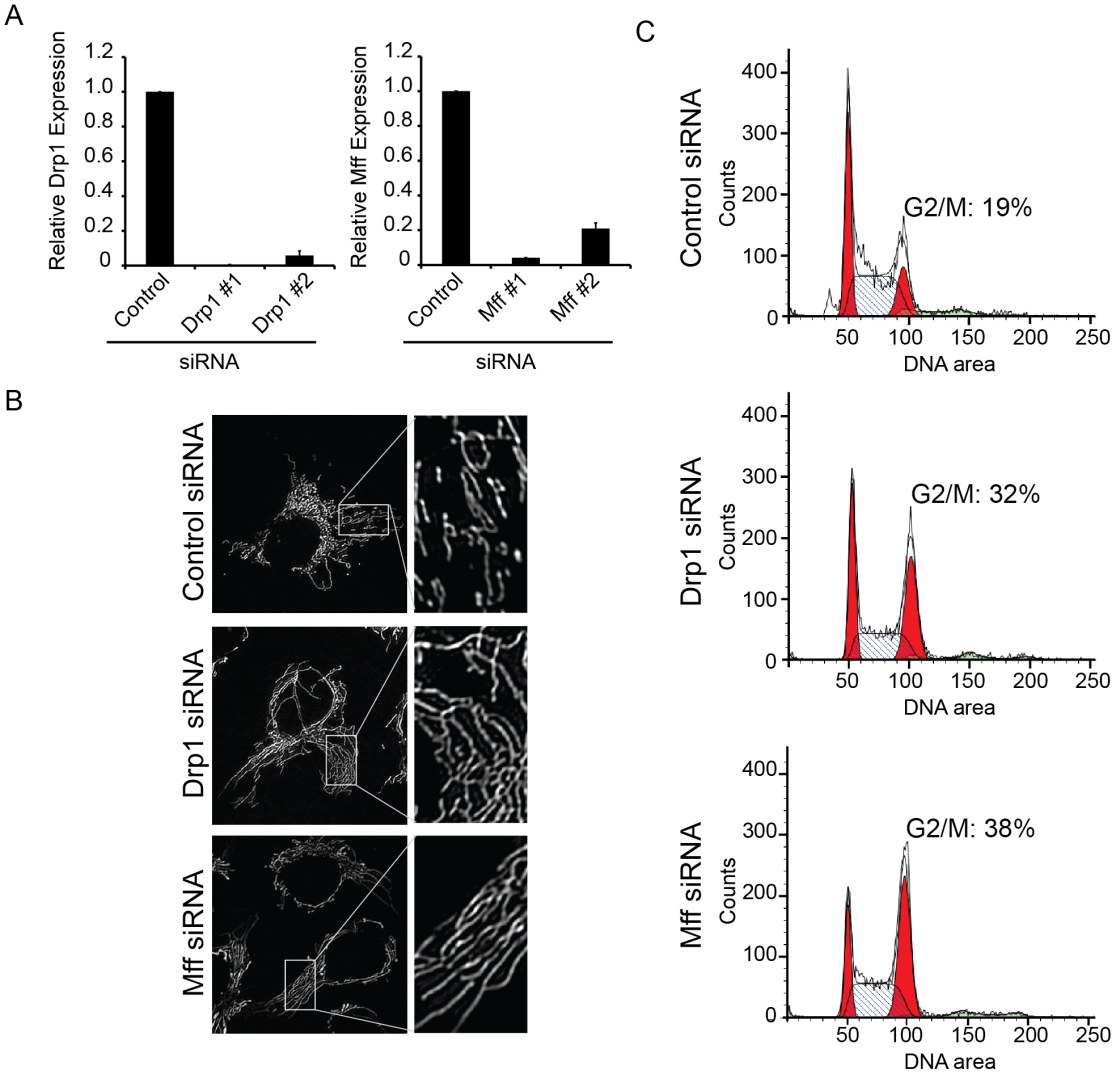
Mitochondrial Hyperfusion Promotes Cellular Accumulation in G2/M. (A) Relative Drp1 and Mff expression (mRNA) was measured by qRT-PCR (relative to HPRT, normalized to control siRNA). Bars represent standard error of replicate experiments. (B) Mitochondrial morphology of U2OS_mitoEYFP following siRNA transfection (48 hours) of mitochondrial fission regulators, Drp1 and Mff compared to control. Insets are 4x magnification of boxed regions. (C) Cell cycle distributions of cells following knockdown of Control, Drp1, or Mff (48 hours) by FACSCaliber analysis of DNA area of propidium iodide-stained cells. The black histogram represents the original cell cycle profile, while the shaded areas represent the fitted cell cycle model following analysis with Modfit LT. The histogram indicates the percentage of cells in G1 (first red peak positioned over 50), G2/M (second red peak positioned over 100), and S phase (the dashed area lying between the two red peaks). Aneuploidy populations are indicated by the green shaded area located along the axis at DNA areas 150 to 200. The percentage of cells in G2/M represents the average (± standard deviation) calculated from two independent experiments.

We investigated whether mitochondrial fission is a requisite step to ensure proper segregation of the mitochondria into two daughter cells by inhibiting mitochondrial fission using siRNA directed against key mitochondrial fission regulators, Mff and Drp1. Depletion of both Drp1 and Mff was confirmed at the RNA level through quantitative real-time PCR (qRT-PCR) (Figure 2A). Targeted knockdown of either Drp1 or Mff resulted in extensive fusion of the mitochondrial reticulum following 48 hours of knockdown as compared to control cells (Figure 2B). Collectively, these data demonstrate that targeted knockdown of distinct mitochondrial fission regulators drives a persisted state of mitochondrial fusion, and that this approach could be used to determine the impact mitochondrial fission on cell cycle progression.

Given the alterations in mitochondrial morphology throughout mitotic cell division, we examined the functional consequence to cell division when mitochondria were held in a persistent state of hyperfusion (Figure 2A). U2OS cells transfected with siRNA against Drp1 or Mff for 48 hours were collected and stained with propidium iodide, a DNA intercalating agent, and analyzed by flow cytometry for DNA content. Compared to control, the cell cycle profiles for both Drp1 and Mff knockdown cells suggested a defect in the ability for cells with hyperfused mitochondria to progress through the cell cycle. Specifically, loss of either Drp1 or Mff resulted in cell accumulation in G2/M as compared to control, indicating that mitochondrial fission is an important feature for cell cycle progression (Figure 2B). Cell cycle quantification revealed that compared to control, cells with mitochondrial hyperfusion showed a 1.7 and 2.0 fold increase block in G2/M for Drp1 and Mff, respectively. The percentage of cells in G2/M for control cells was 19±3.8% compared to 32±2.8% and 38±6.2% for Drp1 and Mff knockdown, respectively.

**Figure 3:**
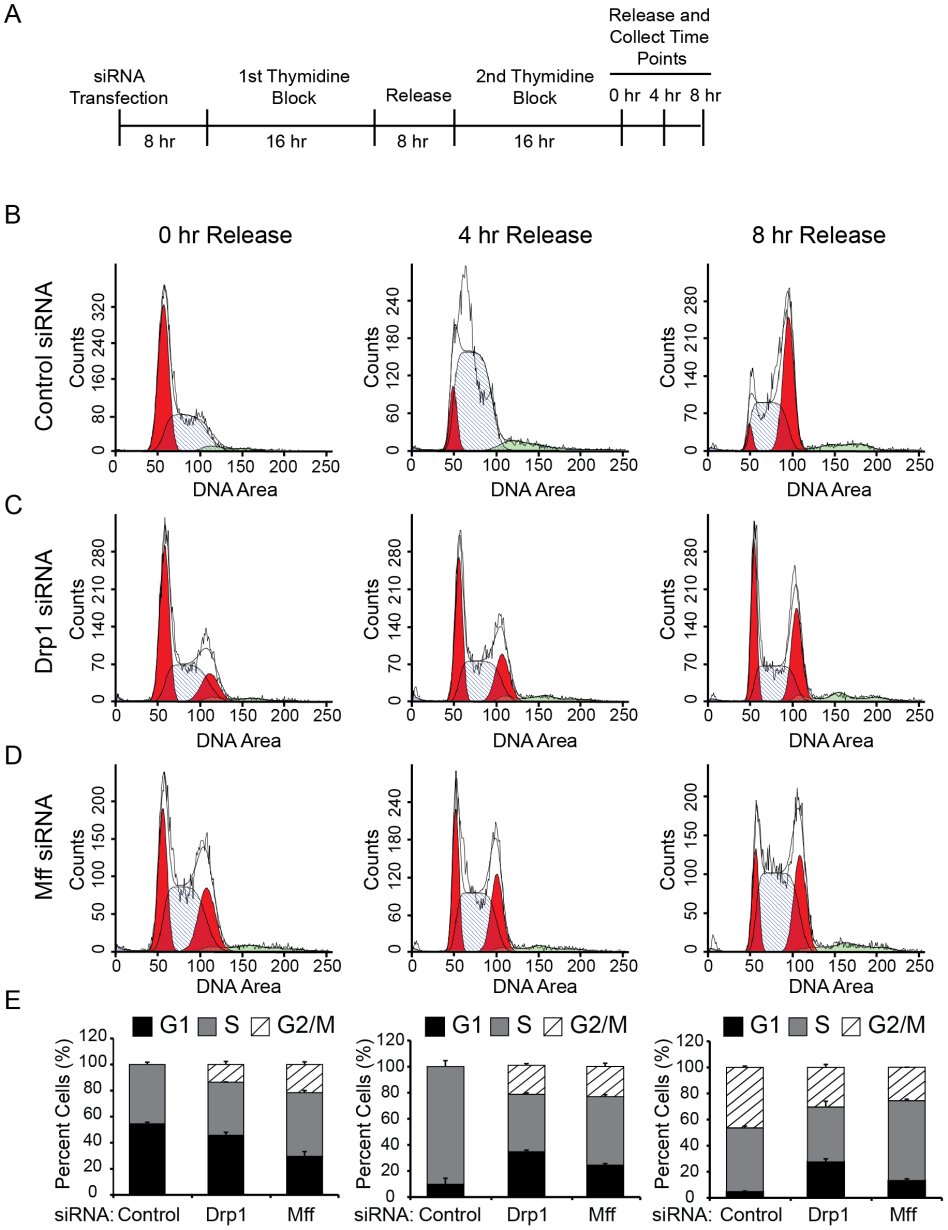
Mitochondrial Hyperfusion Promotes Delayed Progression through the Cell Cycle. (A) Schematic describing the double thymidine block used to synchronize cells in late G1/early S following siRNA transfection. The first collection (0 hour) is timed to occur exactly 48 hours following transfection. (B-D) Cell cycle distributions of cells following release (0, 4, and 8 hour) from double thymidine synchronization for (B) Control, (C) Drp1, and (D) Mff knockdown cells. Cell cycle distributions were obtained following FACSCaliber analysis of DNA area of propidium iodide-stained cells. The histogram indicates the percentage of cells in G1, G2/M and S phase. Aneuploidy populations are indicated by green shaded area located along the axis. (E) Quantification of the percentage of cells in G1, S, or G2/M through the various stages of the cell cycle. Error bars represent standard deviation of the mean from two independent experiments.

The accumulation of cells in G2/M suggests an inability for cells to successfully undergo mitosis when the mitochondrial reticulum is unable to be divided prior to the formation of the daughter cells. To better understand whether the observed G2/M accumulation is the result of either a delay or block in cell cycle progression, we examined cell cycle progression over time following cellular release from a double thymidine block (Figure 3A-D). Thymidine is a pyrimidine deoxynucleoside used to synchronize cells in early S phase[19]. Cell synchronization using a double thymidine block ensures entry into S phase (0−4 hours) and synchronous mitosis (7−8 hours) for > 95% of control cells. U2OS cells were transfected with siRNA against Drp1, Mff or control siRNA before the addition of the first of two thymidine blocks. The transfection timing was staged such that the first cell collection (0 hour release) was taken at 48 hours post transfection (Figure 3A). Collected cells were stained with propidium iodide prior to analysis for DNA content by flow cytometry. As expected, the majority of control cells were stalled in G1 or S following the second thymidine block (Figure 3B. In comparison, cell cycle profiles for both Drp1 and Mff knockdown cells revealed a significant proportion of cells retained in G2/M (Figure 3C-D). Cell cycle profiles 4 hours after release from the thymidine block further confirm that a large proportion of cells with hyperfused mitochondria remain in G2/M compared to control cells (Figure 3B-C). Despite this, a small proportion of cells are able to progress back into G1 (Figure 3B-C) at 8 hours, suggesting the G2/M block is not complete in both Drp1 and Mff siRNA knockdown cells. However, this level of progression back into G1 is at a slower rate compared to control cells. These data suggest that mitochondrial hyperfusion promotes a significant delay in cell cycle progression at the level of G2/M exit and G1 entry (Figure 3E), and is consistent with idea that mitochondrial fission is an important regulatory step in the preparation of the cell for mitotic cell division.

### Persisted mitochondrial hyperfusion sensitizes cells to extrinsic apoptosis

**Figure 4:**
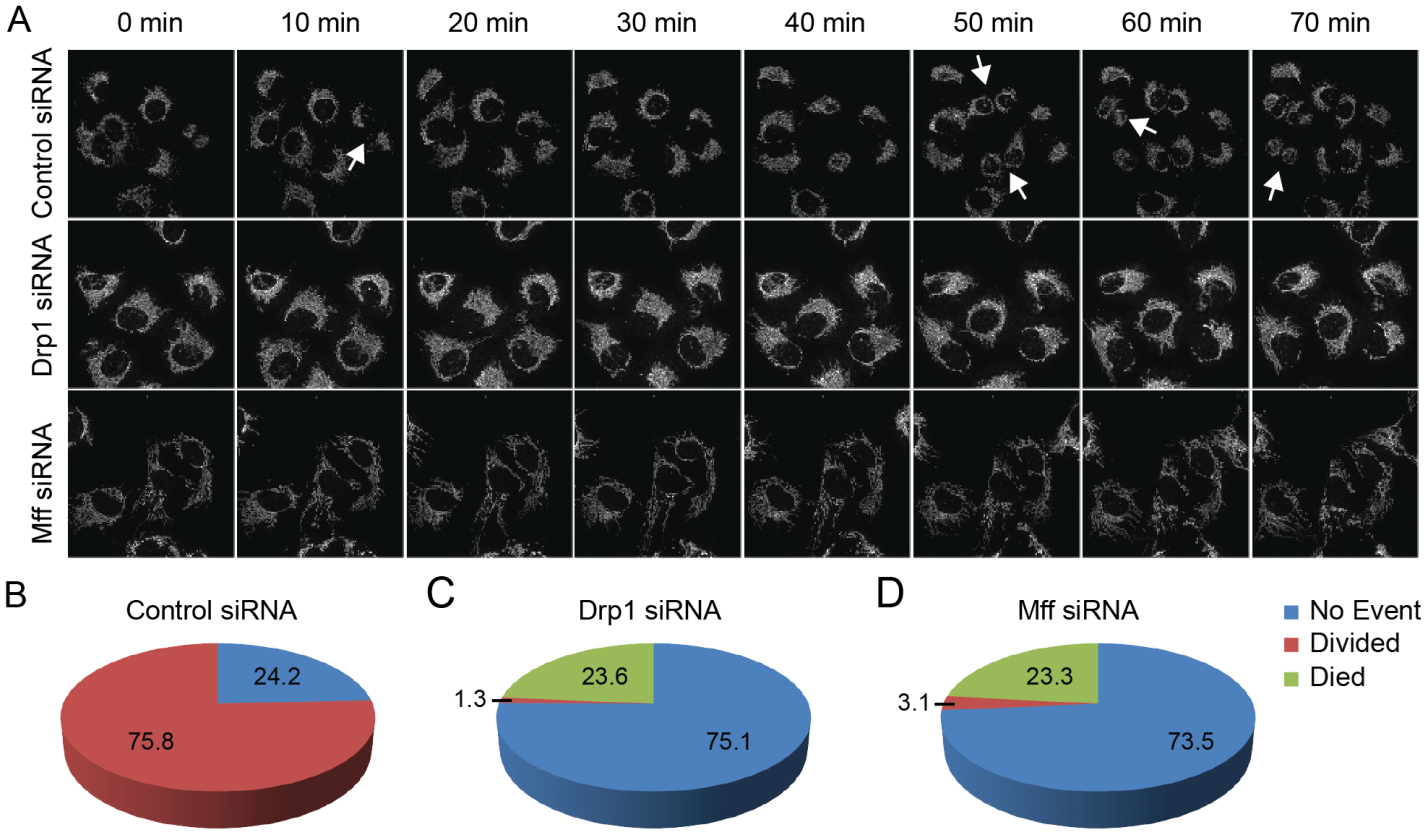
Mitochondrial Hyperfusion Induces Cell Death in addition to Cell Cycle Delay. (A) Time-lapse images tracking U2OS_mitoEYFP for 18 hours following transfection of Drp1, Mff or control siRNAs. Individual cells were tracked throughout the time series to determine if they underwent mitotic cell division events (white arrows). (B) Graphical representation of the percentage of cells in each condition (at least 75 cells tracked for each condition) that underwent a mitotic cell division, underwent cell death (measured by cell retraction, nuclear condensation, and membrane blebbing) or underwent no event.

To better understand the proliferation defect observed in Drp1 and Mff knockdown cells, we tracked individual mitotic events using time-lapse imaging. U2OS_mitoEYFP cells were transfected 48 hours prior to the start of the movie and imaged every 10 minutes for 18 hours. The number of mitotic events were counted and compared to the number of cells that underwent no cellular division for the duration of the movie (Figure 4A). As expected, the number of mitotic events for Drp1 and Mff knockdown cells was significantly lower compared to control cells. In stark contrast to control cells, in which 76% of cells divided at least once during the 18 hour series, mitotic events in Drp1 and Mff knockdown cells were far more rare at 1% and 3% respectively (Figure 4B-D). This decrease in mitotic events supports our previous results (Figures 2 and 3) that mitochondrial hyperfusion results in delayed cell cycle progression.

Interestingly, we found that cells depleted of Drp1 and Mff had a much higher level of cell death than control cell counterparts. Cell death was defined by cells retracting off the plate and demonstrating morphological characteristics of apoptosis such as nuclear condensation and plasma membrane blebbing (Figure 4B-D) [20]. We observed 24% and 23% cell death in Drp1 and Mff knockdown cells respectively, while no cell death events were recorded in control cells. This suggests that these cells may be unable to tolerate extended periods of mitochondrial fusion.

**Figure 5:**
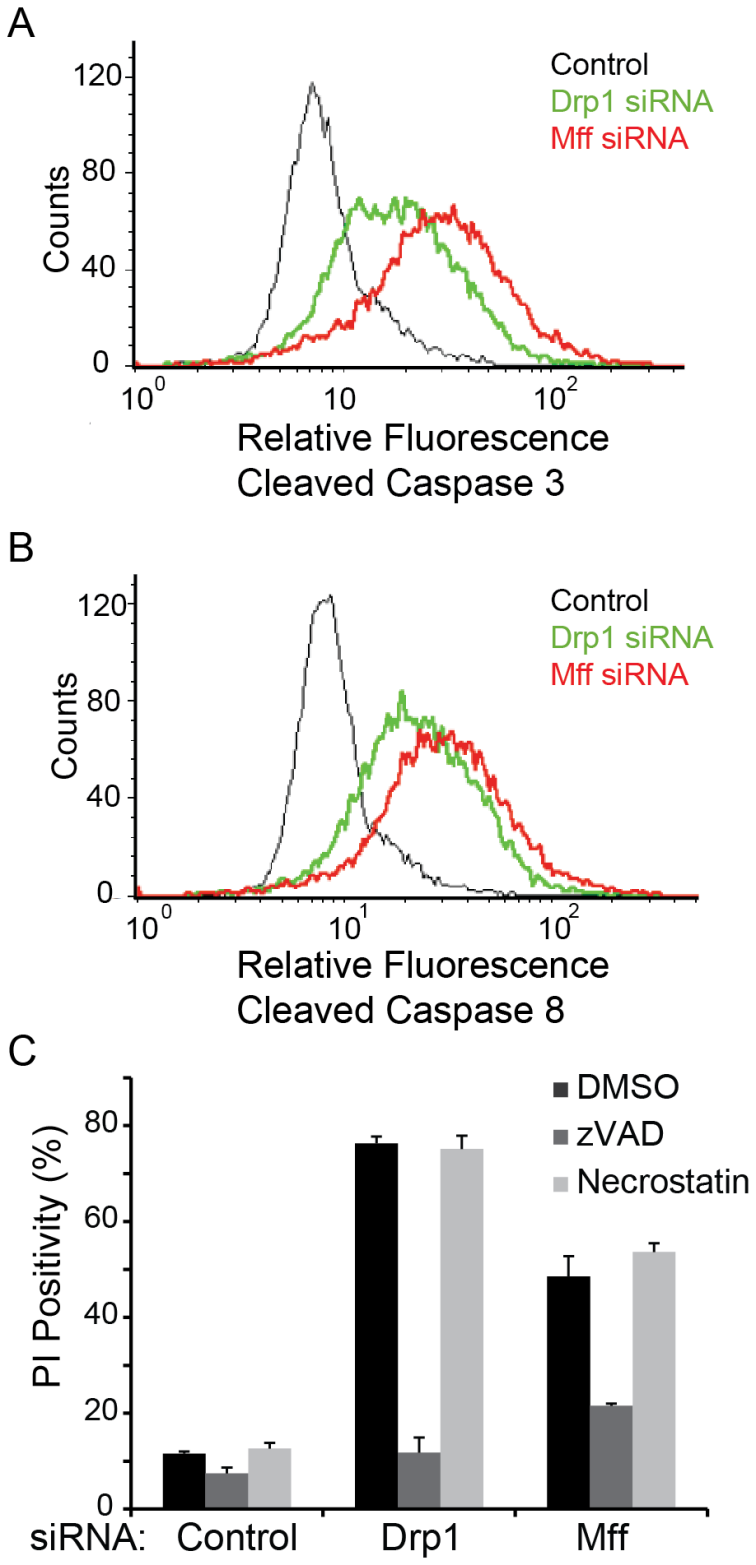
Mitochondrial Hyperfusion Promotes Caspase 8 Dependent Cell Death. (A-C) U2OS cells were transfected with control (black), Drp1 (green), or Mff (red) siRNA for 96 hours. Cells were collected and stained for (A) cleaved caspase 3 or (B) cleaved caspase 8. Positivity of cleaved caspase 3 or 8 is indicated by an increase in fluorescence on the FACS histogram compared to control. (C) U2OS cells transfected with control, Drp1, or Mff siRNAs were treated with pan caspase inhibitor, zVAD (20 μM; dark gray bars), or the necroptosis inhibitor, necrostatin (10 μM; light gray bars), or vehicle (DMSO; black bars) every 24 hours for 96 hours following transfection. Cells were stained with propidium iodide (PI) and percent PI positivity was used as a marker of cell death. Error bars represent standard deviation from two replicate experiments where at least 10,000 events were collected for each treatment.

Replication-induced stress following treatment with chemical compounds such as actinomycin D, nocodazole, or taxol all result in cell cycle arrest and subsequent apoptotic death [21], [22], [23], [24]. We therefore predicted that the observed cell death for Mff or Drp1 knockdown was a result of replicative stress caused by an inability of cells to divide. To determine whether Mff or Drp1 knockdown induces cell death through an apoptotic mechanism, we assayed for the presence of cleaved caspase 3, a known hallmark for apoptotic cell death. Figure 5A demonstrates that 96 hours after siRNA transfection, Mff and Drp1 knockdown cells had elevated levels of cleaved caspase 3 compared to control cells.

In addition to caspase 3 activation, Drp1 and Mff knockdown cells also had elevated levels of activated caspase 8, suggesting that the observed cell death is mediated through a caspase 8 dependent mechanism (Figure 5B). In addition to apoptosis, caspase-8 has been implicated to play a role in the formation of the necroptosome, leading to necroptosis, or programmed necrosis [25], [26]. We confirmed that caspase 8 activation in Mff and Drp1 knockdown cells was not leading to necroptosis, by treating cells with zVAD (a pan caspase inhibitor that blocks apoptosis) or necrostatin-1 (a necroptosis inhibitor that targets RIP1 kinase) [27]. We found that cell death was significantly rescued following treatment of Mff and Drp1 knockdown cells with zVAD at 96 hours post knockdown (Figure 5C). On the other hand, treatment with necrostatin had no significant impact on cell death, suggesting that the observed cell death was not regulated through necroptosis but was instead regulated through caspase activity, specifically caspases 8 and 3. These data together indicate that persistent hyperfusion is toxic to U2OS survival and may be due to the replicative induced stress caused by the inability of cells to fragment and divide their mitochondria prior to mitotic cell division.

## Discussion

Mitotic cell division is an essential cellular process by which eukaryotic cells replicate. A series of checkpoints throughout the stages of the cell cycle ensure that the cell is prepared to divide its components to the two resulting daughter cells. While most of the checkpoints guarantee accurate DNA replication, other checkpoints exist to divide other cellular components and organelles. Here, we have characterized the role of mitochondrial dynamics in mitotic cell division. Specifically, mitochondrial fission defects following targeted loss of two critical mitochondrial fission regulators, Drp1 and Mff, resulted in defects in cell cycle progression. The accumulation of cells in G2/M is consistent with the concept that the mitochondrial network must fragment to ensure the mitochondrial reticulum can be adequately segregated to the two daughter cells. In a recent report by Qian *et al.*, loss of Drp1 caused a G2/M arrest as well as induced replication stress leading to centrosome duplication and chromosomal instability [12]. Interestingly, the G2/M arrest and aneuploidy observed following loss of Drp1 could be reversed through knockdown of cyclin E or ATM (a critical regulator of the DNA damage response). These data suggest that the observed genome instability is induced by replicative stress mediated by Drp1 in an ATM signaling dependent manner [12]. Similar to their findings, our data supports the hypothesis that cell cycle dependent fragmentation of the mitochondria may serve as a novel cellular checkpoint to ensure mitochondrial segregation prior to cytokinesis.

Although the mechanism by which Drp1 initiates coordinated fragmentation of the reticulum prior to cytokinesis remains unknown, one study by Strack *et al*. has strong evidence that posttranslational modifications, specifically phosphorylation, may play a critical role in activating Drp1-dependent fission [28]. Drp1 is regulated at several levels including alternate splicing of the GTPase and variable domains, in addition to a series of post-translation modifications including sumoylatin, S-nitrosylation, O-glycosylation, and phosphorylation [29]. Although the functional impact of each of these forms of Drp1 regulation remains unknown, the authors demonstrated that specific subcellular localization of select Drp1 splice variants to microtubules was dependent on cyclin regulation. Specifically, splice variants including the third but excluding the second alternative axon localized to microtubules but were released into the cytosol from the microtubule bundles following Cdk1 phosphorylation [28]. Cdk1 may therefore be regulating cytoplasmic levels of Drp1 as a mechanistic strategy to induce mitochondrial fission prior to mitotic division.

In addition to the role of mitochondrial fission regulators Drp1 and Mff in cell cycle progression, we found that persistent mitochondrial hyperfusion induced potent apoptotic cell death. Loss of Drp1 or Mff promoted caspase dependent apoptosis, suggesting that mitochondrial fission is an important cellular process to maintain cellular health. Given that cancer cells are inherently reliant on their replicative potential for cellular survival, it is attractive to hypothesize that the cell cycle arrest caused by mitochondrial fusion may sensitize cell survival through replicative stress. Support for the role of mitochondrial dynamics in cancer progression comes from a study in lung cancer, where it was revealed that human lung cancer cell lines have imbalanced expression levels of Drp1/Mfn-2 that promote a state of fragmented mitochondria [30]. Moreover, restoration of mitochondrial fusion through targeted knockdown of Drp1 or overexpression of Mfn-2 resulted not only in a drop in proliferation, but also an increase in apoptotic induction [30]. Inhibition of Drp1 through therapeutic treatment with mdivi-1, a specific Drp1 inhibitor, resulted in significant decrease in murine tumor growth, providing additional support the importance of mitochondrial fission in cancer survival and proliferation [30].

The coordinated action of mitochondrial fission and fusion has critical functions in maintaining mitochondrial function. While the role of mitochondrial dynamics in maintaining mitochondrial health has been well accepted, the role of mitochondrial fission on fusion on cellular fate is less clear. The role of mitochondrial dynamics in cancer cells is even more elusive, as most cancer cell types no longer rely on mitochondrial for energy production and are instead characterized by a glycolytic metabolic profile [3]. Although mitochondria have clear roles in regulating intrinsic apoptosis, we highlight a potential non-canonical function for mitochondrial morphology in regulating cell cycle progression. Given that cancers are hallmarked by their seemingly limitless replicative potential and their relative resistance to apoptosis, finding methods to modulate mitochondrial dynamics in cancer cells may provide new and effective therapeutic targets.
